# The biopsychosocial model between biologism and arbitrariness. A Commentary to H. Helmchen

**DOI:** 10.3389/fpsyg.2014.00126

**Published:** 2014-02-19

**Authors:** Marco Stier

**Affiliations:** Institute for Medical Ethics, History and Philosophy of Medicine, University of MuensterMuenster, Germany

**Keywords:** mental illness, biopsychosocial model, biology, biologism, arbitrariness

In his article “Different conceptions of mental illness: consequences for the association with patients” Helmchen rightly cautions against any kind of dogmatism in psychiatry, regardless of whether it is a social, a psychological or a biological one. Instead, he favors the biopsychosocial model as a remedy for “the narrowing of conceptions that depict only partial aspects of mental illness” (Helmchen, 2013, p. 3). The main criticism of this model is traditionally that it “borders on anarchy” because one can emphasize the “bio” if one wishes, or the “psycho” […], or the “social.” There is “no rationale why one heads in one direction or the other” (Ghaemi, 2009, p. 3). Against this alleged arbitrariness and vagueness of the integrative model Helmchen recommends basing it “on scientifically proven concepts” (Helmchen, 2013, p. 4). Yet, it is not quite clear in which relation the three elements of the integrative model should stand and what its proposed grounding on scientifically proven concepts amounts to. I assume that the biopsychosocial model either has to be based on *biological* facts or else it will remain arbitrary. But if it is based on biological facts—even if not exclusively—it will probably be charged with “biologism” in just the same way as current accounts of biological psychiatry.

It is certainly true that a dogmatic overemphasis of the physiological side of the disorder-coin is ill-advised and in all likelihood to the disadvantage of the patient. Whether biological theories of the mind are in fact utterly brain-focused is, however, a point of contention. Admittedly, there are indeed voices that urge the concept of mental illness to be replaced by an account of brain disease (Bickle, 2006; Akil et al., 2010; Holsboer, 2010; White et al., 2012). But the vast majority of biological psychiatrists *does* try to *understand* the patient's personal situation. Even a hardboiled reductionist cannot avoid asking the respective patient about what she “feels.” The reason is simply that it is up to now impossible to read off the brain whether someone feels depressed or not, whether she has delusions or not. The causes of a mental illness on the one hand and the symptoms on the other are to be found on different levels. This points to the distinction between “explaining” and “understanding” Jaspers is so often cited with and which even a biological psychiatrist cannot—and will not—ignore. In his *General Psychopathology* Jaspers explains:

“The units of phenomenology (e.g., hallucinations, modes of perception, etc.) *are explained by bodily events*. Complex meaningful connections in their turn are considered as units (e.g., a manic syndrome plus all its contents can be regarded as the *effect of a cerebral process* or of some emotional trauma such as the death of an intimate.”) (Jaspers, 1963, p. 305, my italics).

Biological psychiatry is sometimes regarded as nothing but an ideology (Berger, 2001; McLaren, 2010; at least implicitly also Cohen, 1993). On a closer look, things are not that simple. In actual fact, when Helmchen defends the integrative model as a “didactic tool” (Helmchen, 2013, p. 4), this comes quite near even to Bickle who declares within his “ruthless reductionism” that “[h]euristically, higher level investigations and explanations are essential to neuroscience's development” (Bickle, 2006, p. 428). Similarly, Insel and Quirion who demand “that mental disorders be understood and treated as brain disorders” emphasize “the need for a sophisticated understanding of interpersonal relationships along with the use of evidence-based, nonpharmacological treatments” (Insel and Quirion, 2005, p. 2223). Last but not least, Kandel—who, too, is usually regarded a radical reductionist—explains that it “would be unfortunate, even tragic, if the rich insights that have come from psychoanalysis were to be lost in the rapprochement between psychiatry and the biological sciences” (Kandel, 1998, p. 467).

What is, then, the difference between the biological and the biopsychosocial model as Helmchen conceives it? According to the first, mental illness as a phenomenon can be understood on the level of the patient's experiences and be *explained* biologically. According to the latter, the psychological and social levels are essential for an adequate understanding of mental illness, but both should to be based on scientific proven concepts. The much criticized biological psychiatry seems not to be very different from the integrative, scientifically based model.

A critic may object that the searched-for foundation of the biopsychosocial model of mental illness does not at all need to be a biological one. What about Psychology? Isn't it a science, too, with proven concepts? The answer to this objection is—in a nutshell—2-fold. Either psychology is a science or it is not. If it is, it is unavoidable to bring it into accordance with biology, which undoubtedly is a science as well. In cases of theoretical conflict we would need a criterion to decide which concept (or theory, or law) should be given priority. The crucial question, then, is whether the “bio,” the “psycho,” or the “social” should be the maestro in the orchestra of concepts.

If, on the other hand, psychology is not a science, the psychologist will simply not be able to provide the “*scientifically* proven concepts” which are so necessary to mitigate the lurking arbitrariness of the biopsychosocial model of mental illness. It is one thing to use a conception as an auxiliary means for an adequate understanding of something and quite another to make use of it as a scientific foundation.

To sum up, the biopsychosocial model of mental illness is valuable as a reminder that there is more to mental illness than brain functions. Seen as a theory, it will either be based on biology and meet similar trouble as the so called biologism in psychiatry, or else it will indeed be vague and border on anarchy.
